# Effectiveness of N-Acetylcysteine in the Treatment of Renal Deterioration Caused by Long-Term Exposure to Bisphenol A

**DOI:** 10.3390/biom11050655

**Published:** 2021-04-29

**Authors:** Anongporn Kobroob, Wachirasek Peerapanyasut, Sirinart Kumfu, Nipon Chattipakorn, Orawan Wongmekiat

**Affiliations:** 1 Division of Physiology, School of Medical Sciences, University of Phayao, Phayao 56000, Thailand; anongporn.ko@up.ac.th; 2 Renal Physiology Unit, Department of Physiology, Faculty of Medicine, Chiang Mai University, Chiang Mai 50200, Thailand; wachirasek.pee@mahidol.ac.th; 3 Cardiac Electrophysiology Research and Training Center, Department of Physiology, Faculty of Medicine, Chiang Mai University, Chiang Mai 50200, Thailand; bc_nart@hotmail.com (S.K.); nipon.chat@cmu.ac.th (N.C.)

**Keywords:** bisphenol A, kidney, long-term exposure, mitochondria, N-acetylcysteine, oxidative stress

## Abstract

Human health hazards caused by bisphenol A (BPA), a precursor for epoxy resins and polycarbonate-based plastics, are well documented and are closely associated with mitochondrial impairment and oxidative imbalance. This study aimed to assess the therapeutic efficacy of N-acetylcysteine (NAC) on renal deterioration caused by long-term BPA exposure and examine the signaling transduction pathway involved. Male Wistar rats were given vehicle or BPA orally for 12 weeks then the BPA-treated group was subdivided to receive vehicle or NAC concurrently with BPA for a further 4 weeks, while the vehicle-treated normal control group continued to receive vehicle through to the end of experiment. Proteinuria, azotemia, glomerular filtration reduction and histopathological abnormalities caused by chronic BPA exposure were significantly reduced following NAC therapy. NAC also diminished nitric oxide and lipid peroxidation but enhanced renal glutathione levels, and counteracted BPA-induced mitochondrial swelling, increased mitochondrial reactive oxygen species production, and the loss of mitochondrial membrane potential. The benefit of NAC was related to the modulation of signaling proteins in the AMPK-SIRT3-SOD2 axis. The present study shows the potential of NAC to restore mitochondrial integrity and oxidative balance after long-term BPA exposure, and suggests that NAC therapy is an effective approach to tackle renal deterioration in this condition.

## 1. Introduction

Bisphenol A (BPA) is a chemical produced worldwide at a high volume as a starting material for epoxy resins lining food and beverage containers and for the manufacture of polycarbonate plastic products [1,2,3]. The impact of BPA on human health has been called into attention by epidemiological studies revealing that humans are vulnerable to daily BPA exposure and, most importantly, there is a positive correlation between high BPA levels and the incidence of several human diseases such as developmental disorders, cancer, and diabetes [2,4]. These studies encompass both prenatal and postnatal exposure and include several study designs and population types. There is also growing evidence from cell culture along with animal studies in many species, including primates and rodents, showing the damaging effects of BPA on several organs such as the pancreas [5], heart [6], brain [7], liver [8] and reproductive organs [4]. This collective body of evidence provides increasing support that exposure to BPA can be harmful to humans.

The mechanisms of BPA toxicity are likely to be multifactorial and complicated. However, several lines of evidence have demonstrated the depletion of antioxidants as well as the elevation of oxidative injury markers after exposure to BPA [7,8,9] and that oxidative stress is indicated as a key point behind the pathogenesis of BPA. Emerging evidence has also highlighted the involvement of mitochondrial dysfunction in BPA toxicity. BPA has been reported to decrease the enzyme activities of the mitochondrial electron transport chain, decrease ATP synthesis, and increase mitochondrial reactive oxygen species (ROS) generation in rat liver mitochondria [8]. Regarding BPA and the kidney, our recent study has shown that intraperitoneal injection of BPA for 5 consecutive weeks caused kidney damage in rats [10]. Our results also showed the involvement of oxidative stress in the damaging effect of BPA. Most importantly, using an isolated rat kidney mitochondrial model, we provided further evidence to indicate that BPA can act directly at this level causing abnormal mitochondrial function and, finally, whole organ damage [10].

Today, the application of BPA has extended to many consumer products in modern society, putting humans at risk of regular and continuous exposure to BPA. This situation increases the incidence of sustained renal degeneration and can eventually lead to end-stage renal disease. Epidemiological data provided further support, since an association of high urinary BPA and low-grade albuminuria was observed in both adults and children [11,12]. Finding strategies to overcome the effects of long-term BPA exposure on the kidneys is therefore essential.

N-acetylcysteine (NAC) is generally recognized as an inexpensive human mucolytic drug. It also has many pleiotropic effects including antioxidant, anti-inflammation, and anti-apoptosis effects [13]. Studies have shown the therapeutic efficacy of NAC in a variety of oxidant-mediated disorders in many organ systems [14]. In terms of the kidney, there is substantial evidence demonstrating the effectiveness of NAC in protection against several kidney injury models including renal ischemia-reperfusion [13], sepsis [15] and, particularly, nephrotoxicity [14]. Clinically, NAC is accepted as a compound for toxicity prevention, with a very low rate of adverse events, in certain scenarios such as contrast-induced nephropathy [16].

The protective role of NAC on BPA toxicity via its antioxidant power has recently been reported in various organ systems such as brain [7] and testis [17]. Consistent with these studies, experiments using a BPA-treated cell line also demonstrated that cell viability was significantly increased when treated with NAC and the improvement of oxidative stress is thought to be a major mechanism behind these effects [18]. Importantly, there are also increasing research data supporting the advantage of NAC as an effective mitochondrial protective agent apart from its well-recognized powerful antioxidant effects [19]. As a high risk of persistent BPA exposure together with oxidative stress and mitochondrial injury contribute significantly to the pathogenesis of BPA toxicity, it is interesting to investigate whether NAC could be an effective therapy to suppress adverse renal consequences after long-term BPA exposure. The signaling pathway associated with renal alterations and the potential of NAC in this condition was also explored.

## 2. Materials and Methods

### 2.1. Animal Preparations

Twenty-four male Wistar rats (130–150 g) obtained from Nomura Siam International, Bangkok, Thailand, were housed under standard temperature (24 ± 1 °C) and humidity (55 ± 5%) conditions, with a 12 h light-dark cycle, food and water given ad libitum. This study was approved by the Institutional Animal Care and Use Committee at the Faculty of Medicine, Chiang Mai University (Project number 38/2561) in compliance with the guidance for the use of animals by the National Research Council of Thailand.

### 2.2. Experimental Designs

One week after acclimatization, rats were initially assigned to receive vehicle (corn oil) (*n* = 8) or BPA (50 mg/kg/day) (*n* = 16) via oral gavage for 12 weeks. At the end of week 12, the vehicle-treated control group continued to receive vehicle for further 4 weeks, while rats treated with BPA were randomly allocated to receive different treatments, i.e., vehicle or NAC (100 mg/kg/day, orally) (*n* = 8 each), in addition to BPA for a further 4 weeks. The dose of BPA was based on previous study showing the adverse health effect of BPA exposure on the kidney [10], while the dose of NAC was selected according to its protection against BPA-induced cognitive dysfunction in rats [7].

Body weight, food and water intake were recorded every day. At the end of experiments (week 16), rats were placed in metabolic cages for 24 h urine collections. Blood and kidney samples were taken thereafter under thiopental anesthesia (80 mg/kg, i.p.) for evaluation of renal function, oxidative stress, mitochondrial function, and histopathology.

### 2.3. Biochemical Assays

#### 2.3.1. Evaluations of Renal Functions

Blood urea nitrogen (BUN), serum creatinine, urine creatinine and urine protein were assayed using an automatic analyzer (Beckman Coulter, Inc., Brea, CA, USA). Creatinine clearance and urine protein-to-creatinine ratio were calculated.

#### 2.3.2. Evaluation of Renal Oxidative Stress

The renal tissues were homogenized in an appropriate buffer using a Potter Elvehjem glass-teflon homogenizer (Wheaton Science, Millville, NJ, USA). The tissue homogenates were centrifuged at 10,000× *g* for 15 min at 4 °C, and the supernatant was collected for oxidative stress assays. Lipid peroxidation was estimated in terms of malondialdehyde (MDA) using TBARS assay kit (Cayman Chemical, Ann Arbor, MI, USA) according to the manufacturer’s instruction. Nitric oxide (NO) and reduced glutathione (GSH) were assayed using commercially available kits (Bioassay Systems, Hayward, CA, USA).

### 2.4. Histopathological Studies

Kidney tissues fixed in 10% neutral buffered formaldehyde were routinely processed for light microscopic studies as described earlier [20]. The 4 μm paraffin sections were stained with Hematoxylin and Eosin (H&E) and examined under a Leica DM750 photomicroscope (Leica Microsystems, Heerbrugg, Switzerland). For electron microscopic studies, renal cortical tissues were fixed overnight with 2.5% glutaraldehyde in 0.1 M phosphate buffer (pH 7.4, 4 °C) and processed according to the previously published method [20]. Sections of 60–80 nm, stained with uranyl acetate and lead citrate, were examined using a JEM-2200 FS transmission electron microscope (JEOL, Tokyo, Japan).

### 2.5. Mitochondrial Studies

#### 2.5.1. Preparation of Mitochondrial Proteins

Kidney tissues were homogenized in cold lysis buffer (230 mM mannitol, 70 mM sucrose, 1 mM EDTA, and 10 mM Tris-HCl, pH 7.4) and mitochondria were isolated by differential centrifugation as previously described [10]. The final mitochondrial pellets were suspended in ice-cold respiration buffer containing 250 mM sucrose, 5 mM KH_2_PO_4_, 10 mM Tris-HCl, 2 mg/mL BSA, pH 7.2. A bicinchoninic acid (BCA) assay was used to quantify protein content of the mitochondria, and bovine serum albumin was used as standard.

#### 2.5.2. Determination of Mitochondrial Reactive Oxygen Species (ROS) Production

Mitochondrial ROS were assayed using a cell-permeable fluorogenic probe 2′,7′-dichloro-fluorescein diacetate (DCFDA) as previously described [10]. Briefly, mitochondria were incubated at 25 °C with 2 µM DCFDA for 60 min. The fluorescence emission from DCF was determined by fluorescence microplate reader with excitation and emission spectra set at 485 nm (bandwidth 10 nm) and 530 nm (bandwidth 5 nm), respectively. The ROS levels were expressed as arbitrary units of fluorescence intensity of DCF.

#### 2.5.3. Determination of Mitochondrial Membrane Potential

Mitochondrial membrane potential (ΔΨm) was assessed using a lipophilic cationic fluorescence JC-1 dye according to the method described previously [10]. Briefly, mitochondria were stained with 310 nM JC-1 and incubated at 37 °C for 30 min. The red J-aggregate and green monomer forms of JC-1 were detected by a fluorescence microplate reader at an excitation/emission of 485/590 and 485/530 nm, respectively. The ratio of red/green fluorescence intensity was calculated and used to indicate the change in ΔΨm, where a decrease in the ratio reflected mitochondrial membrane potential dissipation.

#### 2.5.4. Determination of Mitochondrial Swelling

Mitochondrial swelling was determined by measuring the change in absorbance of mitochondria at 540 nm over 15 min [10]. Kinetic measurements were carried out every 1 min at 25 °C using a microplate reader (Synergy^TM^ H4, BIOTEK^®^ Instruments, Inc., Winooski, VT, USA). A decrease in absorbance indicates mitochondrial swelling.

### 2.6. Western Blot Analysis

Renal cortical tissues were extracted in buffer containing 20 mM Tris pH 6.8, 1 mM sodium orthovanadate, 5 mM sodium fluoride, and protease inhibitor. The supernatants were collected, and protein content was measured using a Bradford protein assay kit (Bio-Rad Laboratories, Hercules, CA, USA). The protein lysates were mixed with loading buffer and separated by 10% SDS-PAGE under denaturing conditions then transferred onto nitrocellulose membranes (Thermo Fisher Scientific, Waltham, MA, USA). The membranes were blocked with either 5% skim milk or 5% bovine serum albumin in Tris-buffered saline containing 0.1% Tween (TBST) and then incubated overnight at 4 °C with primary antibodies against adenosine monophosphate-activated protein kinase (AMPK), acetylated superoxide dismutase 2 (Ac-SOD2), superoxide dismutase 2 (SOD2), sirtuin 3 (SIRT3), pro-caspase 3, cleaved-caspase 3, B-cell lymphoma-2 (Bcl-2), (Cell Signaling Technology, Danvers, MA, USA), phosphorylated AMPK (p-AMPK^Thr172^), peroxisome proliferator-activated gamma receptor coactivator-1α (PGC-1α) (Millipore Corporation, MA, USA), Bcl-2-associated X protein (Bax) and β-actin (Santa Cruz Biotechnology, Santa Cruz, CA, USA) followed by the appropriate horseradish peroxidase (HRP)-conjugated second antibodies (Millipore Corporation, MA, USA) for 1 h at room temperature. Detection of protein was performed using Bio-Rad’s Clarity ECL Western Blot Substrate kits and the blot bands were developed in ChemiDoc Touch Imaging System (Bio-Rad Laboratories, Inc., Philadelphia, PA, USA). Analysis of protein expression was determined by the Image J program [21]. Each protein expression was normalized with β-actin expression.

### 2.7. Statistical Analysis

Data are expressed as mean ± SEM. One-way analysis of variance (ANOVA) followed by Fisher post-hoc test or nonparametric Kruskal Wallis test (as appropriate) was used to determine differences between the groups. *p* < 0.05 was considered statistically significant. All analyses were performed using SPSS software version 20.0 (SPSS Inc., Chicago, IL, USA).

## 3. Results

### 3.1. Effects of Long-Term BPA Exposure and NAC Treatment on Body Weight, Kidney Weight, Food and Water Intake

Body weight at the start of experiment was very similar in all groups studied (Table 1). After 12 weeks of continuous BPA exposure, all BPA-exposed rats experienced less body weight gain than vehicle-fed rats, although the intake of food and water was almost the same. In addition to the lower body weight, BPA-treated rats also experienced a significant decrease in absolute kidney weight at the end of week 16 compared with vehicle-controlled rats. However, no significant differences were detected in terms of kidney weight to body weight ratio. NAC administration to BPA-exposed rats from week 12 to week 16 had no measurable effect on the change of these parameters caused by BPA.

### 3.2. Effects of Long-Term BPA Exposure and NAC Treatment on Renal Function and Histopathology

**Figure 1:**
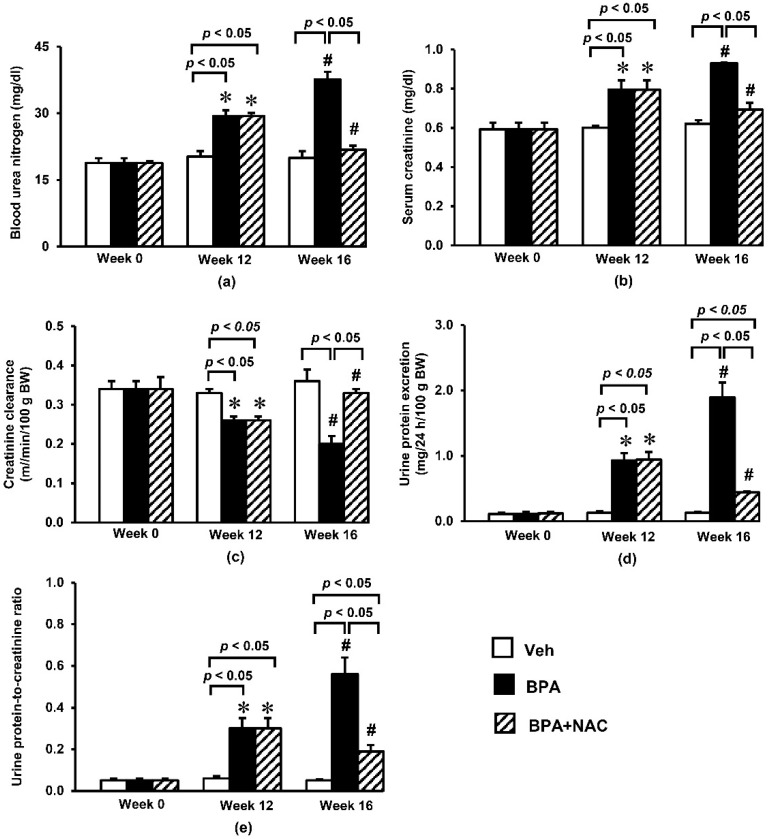
Effects of long-term BPA exposure and NAC treatment on (**a**) blood urea nitrogen; (**b**) serum creatinine; (**c**) creatinine clearance; (**d**) urine protein excretion; (**e**) urine protein-to-creatinine ratio. Values are mean ± SEM (*n* = 6 each). Veh: vehicle-treated group; BPA: BPA-treated group; BPA + NAC: BPA plus NAC-treated group. * *p* < 0.05 vs. their respective values in week 0. ^#^ *p* < 0.05 vs. their respective values in week 12.

Exposure to BPA for a period of 12 weeks resulted in significant increases in blood urea nitrogen (Figure 1a) and serum creatinine (Figure 1b) compared to the vehicle control. The levels of urea nitrogen and creatinine progressively increased and reached about 99% and 58% above their baseline values, respectively, by the end of experiment. Consistent with the accumulations of blood urea nitrogen and serum creatinine, BPA exposure caused an obvious reduction (*p* < 0.05) in creatinine clearance by some 24% and 41% after 12 and 16 weeks of exposure, respectively (Figure 1c).

In addition, a remarkable increase (*p* < 0.05) in urine protein excretion of about nine-fold from the respective baseline values was observed at week 12 following BPA exposure, and reached approximately 19-fold within week 16 (Figure 1d). Calculation of urine protein-to-creatinine ratio (Figure 1e) exhibited similar trend to the 24 h urine protein excretion. It was found that the urine protein-to-creatinine ratio dramatically rose from its baseline value in week 0 to about six-fold in week 12, and further increased to 11-fold in week 16. Interestingly, NAC treatment showed strong potential to significantly ameliorate all renal functional alterations caused by oral BPA exposure for 16 consecutive weeks.

**Figure 2:**
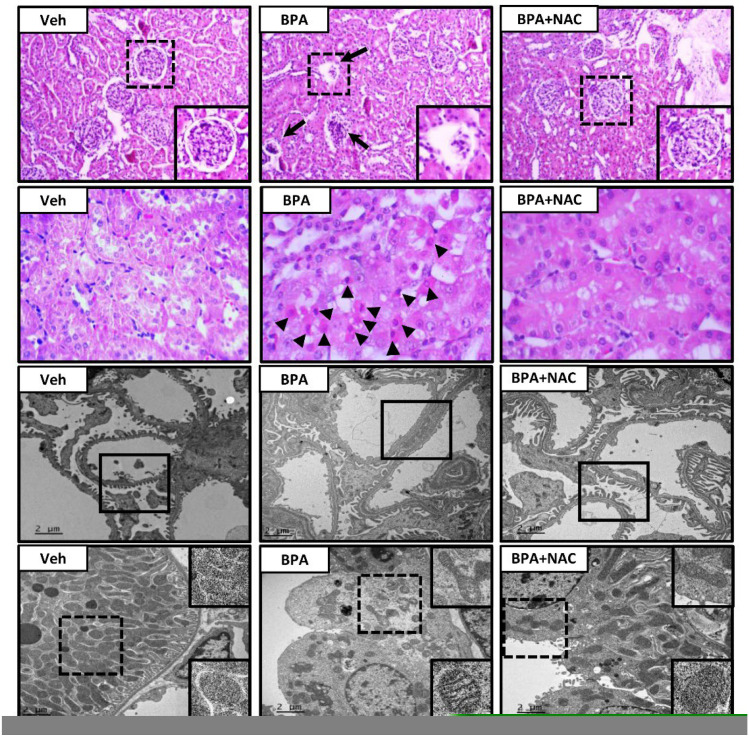
Histopathological changes following long-term BPA exposure and NAC treatment. The first and second panels show kidney sections stained with hematoxylin and eosin (H&E, 10× and 40×, respectively). The third and last panels show transmission electron micrographs of glomerulus and renal tubules, respectively (Original magnification: 3000×). Veh: vehicle-treated group; BPA: BPA-treated group; BPA + NAC: BPA plus NAC-treated group. Arrows and arrowheads show abnormal glomerulus and apoptotic cells, respectively. The inserted frame is enlarged from the dashed area. The squares within the third panel highlight the morphology of the podocytes, especially the podocyte effacement in the BPA-treated group.

Light and electron microscopic examination following long-term BPA exposure and NAC treatment are depicted in Figure 2. The vehicle-control rat exhibited all normal characteristics of glomerulus (first panel), renal tubules (second panel), podocytes (third panel), and mitochondria (lower panel). In contrast, H&E-stained kidney section from the long-term BPA-exposed rat demonstrated glomerular structural changes with some of them becoming atrophied. A large number of apoptotic cells were found in the proximal tubules and distributed throughout the kidneys. Consistent with light microscopy, podocyte effacement characterized by flattening, widening, and shortening of the foot processes, and reduction in the frequency of filtration slits were detected from electron photomicrographs of the long-term BPA-exposed group. Besides, mitochondria within the proximal tubule appeared swollen, fragmented, with disrupted cristae, and decreased in the number. The morphological changes induced by BPA were significantly diminished upon treatment with NAC for 4 weeks.

### 3.3. Effects of Long-Term BPA Exposure and NAC Treatment on Renal Oxidative Stress and Mitochondrial Function

**Figure 3:**
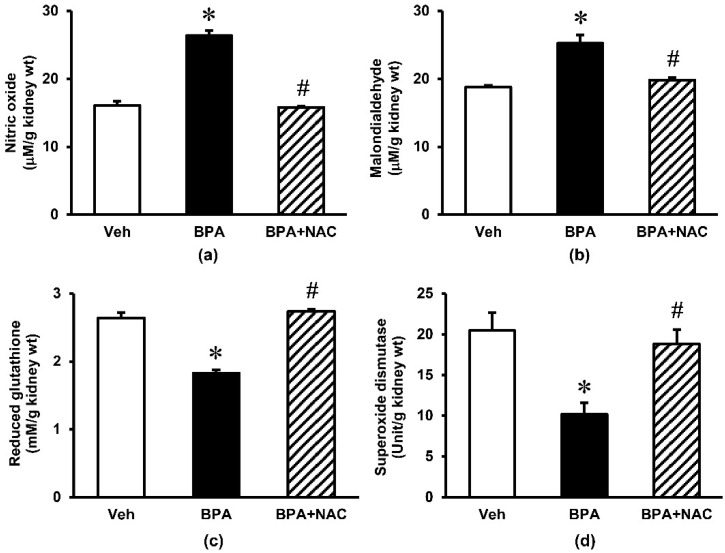
Effects of long-term BPA exposure and NAC treatment on renal oxidative stress. (**a**) nitric oxide; (**b**) malondialdehyde; (**c**) reduced glutathione; (**d**) superoxide dismutase. Values are mean ± SEM (*n* = 6). Veh: vehicle-treated group; BPA: BPA-treated group; BPA + NAC: BPA plus NAC-treated group. * *p* < 0.05 vs. Veh, ^#^ *p* < 0.05 vs. BPA.

Repeated exposure to BPA for 16 weeks produced an obvious increase (*p* < 0.05) in the kidney tissue levels of nitric oxide and malondialdehyde by some 64% and 25%, respectively (Figure 3). These increments were significantly blunted upon treatment with NAC during the last 4 weeks of BPA exposure. The non-enzymatic antioxidant glutathione as well as enzymatic antioxidant superoxide dismutase were also reduced significantly following long-term BPA exposure. Again, NAC treatment was able to restore these changes to the values that were comparable to control.

**Figure 4:**
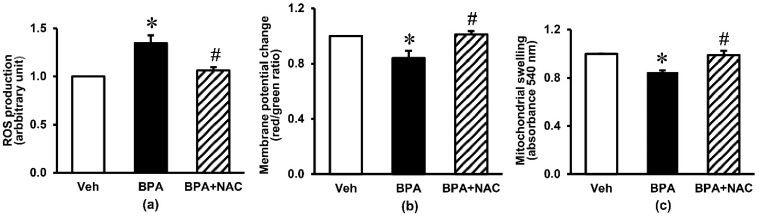
Effects of long-term BPA exposure and NAC treatment on renal mitochondrial function. (**a**) ROS production; (**b**) membrane potential change; (**c**) mitochondrial swelling. Values are mean ± SEM (*n* = 6). Veh: vehicle-treated group; BPA: BPA-treated group; BPA + NAC: BPA plus NAC-treated group. * *p* < 0.05 vs. Veh, ^#^ *p* < 0.05 vs. BPA.

Figure 4 demonstrates the kidney mitochondrial function in response to long-term BPA exposure and NAC treatment. There was a considerable increase in mitochondrial ROS production of about 30% (*p* < 0.05) in the BPA-exposed group compared with the vehicle control group. Exposure to BPA for 16 weeks also produced a fall in mitochondrial membrane potential change (*p* < 0.05) and a swelling of mitochondria as reflected by a significant decrease in mitochondrial absorbance. Treatment with NAC concurrently with BPA, started at week 12 and continued through to the end of week 16, showed a remarkable protection against mitochondrial functional alterations induced by long-term BPA exposure (*p* < 0.05).

### 3.4. Effects of Long-Term BPA Exposure and NAC Treatment on Renal Cortical Expressions of Apoptotic Markers

**Figure 5:**
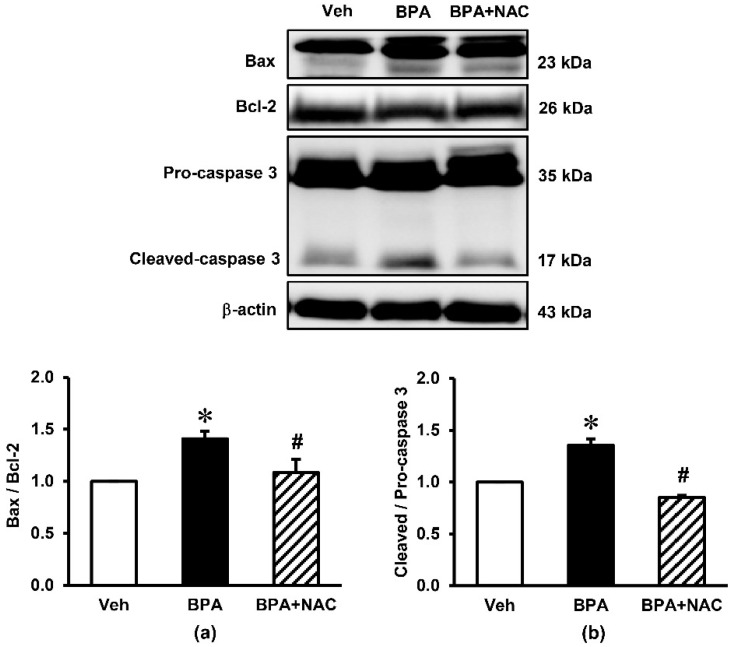
Effects of long-term BPA exposure and NAC treatment on renal cortical expressions of apoptotic markers. (**a**) Bax/Bcl-2; (**b**) Cleaved/Pro-caspase-3. Values are mean ± SEM (*n* = 4). Veh: vehicle-treated group; BPA: BPA-treated group; BPA + NAC: BPA plus NAC-treated group. * *p* < 0.05 vs. Veh, ^#^ *p* < 0.05 vs. BPA.

The impact of long-term BPA exposure and NAC treatment on apoptosis of the kidney are presented in Figure 5. Western blotting analysis showed that the protein level of pro-apoptotic Bax was significantly increased after long-term BPA exposure but significantly decreased after the administration of NAC. By contrast, BPA decreased the expression of anti-apoptotic Bcl-2 protein compared to vehicle (*p* < 0.05), while this reduction was restored upon concurrently treatment with NAC. The ratio of cleaved-caspase 3/pro-caspase 3 was also increased after exposure to BPA for 16 weeks (*p* < 0.05), which was suppressed by NAC treatment.

### 3.5. Effects of Long-Term BPA Exposure and NAC Treatment on Renal Cortical Expressions of Signaling Proteins Involved in AMPK-SIRT3-SOD2 Axis

**Figure 6:**
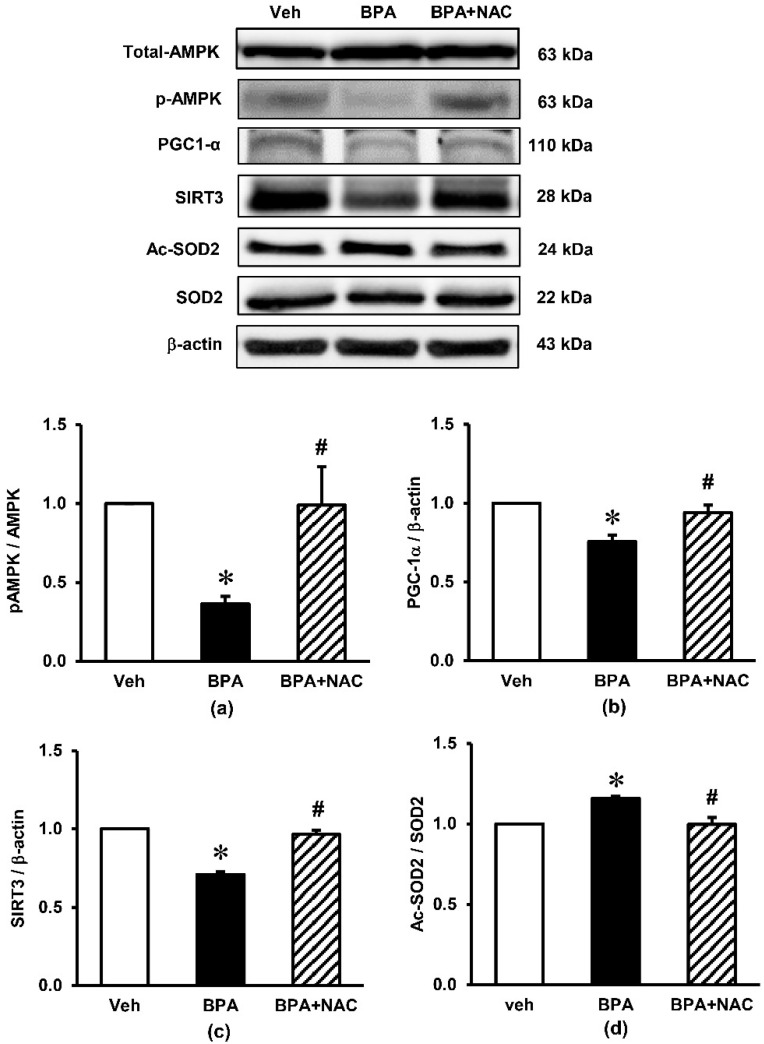
Effects of long-term BPA exposure and NAC treatment on renal cortical expressions of signaling proteins involved in the AMPK-SIRT3-SOD2 axis. (**a**) pAMPK/AMPK; (**b**) PGC-1α/β-actin; (**c**) SIRT3/β-actin; (**d**) Ac-SOD2/SOD2. Values are mean ± SEM (*n* = 4). Veh: vehicle-treated group; BPA: BPA-treated group; BPA + NAC: BPA plus NAC-treated group. * *p* < 0.05 vs. Veh, ^#^ *p* < 0.05 vs. BPA.

The expression of signaling proteins involved in the AMPK-SIRT3-SOD2 axis, which is the key pathway involved in the regulation of mitochondrial biogenesis and the maintenance of homeostasis in the cells, are shown in Figure 6. Although long-term BPA exposure had no effect on the expression of AMPK total protein, it markedly decreased the phosphorylation of AMPK (p-AMPK), resulting in a robust decrease in the ratio of p-AMPK/AMPK (*p* < 0.05) compared to the vehicle control. A significant decrease in PGC-1α as well as SIRT3 was observed after the rats were exposed to BPA for 16 consecutive weeks. In addition, BPA exposure significantly enhanced the expression of Ac-SOD2 while decreasing the expression of SOD2, causing an increase in the Ac-SOD2/SOD2 ratio (*p* < 0.05). All these changes caused by BPA were significantly inhibited when the BPA-exposed rats were treated with NAC.

## 4. Discussion

This study evaluated the impacts of long-term exposure to BPA on the kidney with particular attention paid to the potential therapeutic role of NAC in this condition. The study outcomes provide compelling evidence indicating the effectiveness of NAC in impeding the renal deterioration caused by BPA through the protection of mitochondria.

Exposure to BPA for 16 consecutive weeks leads to a deterioration of kidney function as signified by a reduction in creatinine clearance together with the retention of blood urea nitrogen and serum creatinine progressively over time. The development of renal functional impairment in the current study is potentially a consequence of the actions of BPA on both glomerular and tubular sites. The glomerular effect of BPA is suggested based on the presence of proteinuria (increased urine protein excretion including urine protein-to-creatinine ratio), abnormal glomerular shape, and the characteristic of podocyte effacement; while the manifestation of apoptotic cells in the proximal tubules coupled with structural deformity of mitochondria observed from light and electron microscopies reflect the influence of BPA in renal tubular damage. Our results are compatible with an earlier report showing BPA induced podocytopathy and proteinuria in mice including a diminished expression of the slit diaphragm proteins nephrin and podocin in cultured podocytes [22]. In addition, a study reports degeneration of the proximal tubule epithelium and symptoms of cell’s nucleus pyknosis after BPA exposure [23]. In accordance with histopathological studies, we also detected increasing expressions of Bax, decreasing Bcl-2, and increasing cleaved caspase-3/caspase-3 in the kidney tissues after 16 weeks of BPA exposure. As these proteins are important regulators and mediators of the mitochondrial apoptotic pathway [24,25], it is believed that the impairment of renal function after long-term BPA exposure is the result of mitochondrial injury caused by BPA. Evidence obtained from the current study in which renal mitochondrial dysfunction was shown by increased mitochondrial ROS production, decreased mitochondrial membrane potential, and mitochondrial swelling, provided further support to this suggestion.

In recent years, the direct role of BPA on mitochondria has been revealed. The study using both freshly isolated hepatocytes as well as liver mitochondria isolated from BPA-treated rats showed that the dose-dependent induction of hepatotoxicity was produced by enhancing oxidative stress and impairing complex I and III enzyme activities of the mitochondrial electron transport chain [8]. Regarding the kidney, our previous study using an in vitro model was able to demonstrate a dose-dependent impairment in mitochondrial function when BPA was applied directly onto the isolated kidney mitochondria [10]. This in vitro experiment further pointed out that BPA-induced mitochondrial oxidative stress, as exhibited by increasing the levels of mitochondrial MDA and decreasing antioxidant glutathione, is the causative factor of mitochondrial dysfunction and leads to whole organ damage.

Interestingly, the present investigation revealed that treatment with NAC for 4 weeks was able to offset BPA-induced renal deterioration, as seen by the improvements in azotemia, proteinuria, mitochondrial functional impairment, renal structural changes, and the suppression of all apoptotic protein expressions. The effectiveness of NAC was evident even though it was given after BPA exposure for 12 weeks, when the renal damage has already been established. The benefit of NAC was observed in association with the restorations of all oxidative indexes (NO, MDA, GSH, and particularly SOD) within the kidneys, suggesting that NAC exerted its renoprotection via its redox control properties. This is compatible with several publications showing the free radical scavenging and antioxidant properties of NAC [26,27,28,29].

An additional significant finding in the present study is the decreased expressions of p-AMPK/AMPK ratio, PGC-1α, SIRT3, SOD2 and the increased expression of Ac-SOD2 after 16 weeks of BPA exposure, while treatment with NAC restored these alterations. The AMPK-PGC-1α-SIRT3-SOD2 axis has been recognized as essential for the regulation of mitochondrial oxidative stress and biogenesis [30]. SIRT3, the primary mitochondrial NAD^+^-dependent protein deacetylase, plays a key role in maintaining mitochondrial redox homeostasis by regulating the function of electron transport chain complexes I and III and thereby prevents ROS generation within the mitochondria [30,31]. It also contributes to ROS detoxification by deacetylation of SOD2 and, as a result, activation of mitochondrial antioxidant enzyme SOD2 [32]. Based on our findings, it is most likely that the upregulation of SIRT3 after NAC supplementation is a crucial mechanism responsible for the improvement of mitochondrial injury induced by long-term BPA exposure. Studies in various experimental models also show that upregulation of SIRT3 reduces mitochondrial injury and preserves organ function, while deletion of SIRT3 exacerbates the injury [30,33,34,35].

Study in vitro using muscle cells and hepatocytes showed that knockdown PGC-1α gene led to decreased SIRT3 gene expression, and knockdown SIRT3 decreased the stimulatory effect of PGC-1α on mitochondrial biogenesis [36]. The study concluded that SIRT3 functions as a downstream target gene of PGC-1α and is essential for PGC-1α-dependent induction of ROS-detoxifying enzymes and several components of the mitochondrial respiratory chain [36]. There are also reports of AMPK-activated PGC-1α in the regulation of mitochondrial biogenesis and oxidative stress [36,37]. A study of a myocardial ischemia/reperfusion model demonstrated that inhibition of AMPK significantly reduced the expressions of PGC-1α and SIRT3, impaired mitochondrial function, increased mitochondrial oxidative damage, and promoted myocardial injury [30]. In addition, BPA-induced toxicity has previously been shown to be related to the disruption of ETC and decreased ATP production [8]. Based on these available data, together with the crucial role of AMPK in the maintenance of ATP balance, it is possible that NAC may protect against BPA nephrotoxicity in the present study via its ability to facilitate ATP production and trigger AMPK phosphorylation and activation, which subsequently activate SIRT3 activity and preserve mitochondrial homeostasis. As AMPK as well as PGC-1α is upstream to SIRT3, further investigation is required to identify the definite site of action of NAC in this circumstance. However, the findings obtained herein underscore the therapeutic efficacy of NAC to cope with long-term BPA induced renal deterioration, at least in part, through an activation of the AMPK-PGC-1α-SIRT3-SOD2 axis.

## 5. Conclusions

The present outcomes confirm the deleterious consequences of long-term BPA exposure on the kidney and provide additional evidence to support that the disturbance of mitochondrial homeostasis, via AMPK-PGC-1α-SIRT3-SOD2 axis, is a key mediator in BPA-induced nephrotoxicity. Above all, emerging evidence from this study points towards NAC as an effective therapeutic remedy for this disorder.

## Figures and Tables

**Table 1:** Effects of long-term BPA exposure and NAC treatment on body weight, kidney weight, food and water intake.

Parameters		Week 0			Week 12			Week 16	
	Veh	BPA	BPA+NAC	Veh	BPA	BPA + NAC	Veh	BPA	BPA + NAC
BW (g)	135.0	135.0	134.0	459.17	415.00	415.83	466.67	396.67	395.00
	±1.83	±0.62	±1.53	±5.07 ^‡^	±14.61 *^,‡^	±10.12 *^,‡^	±10.54	±4.94 *	±10.17 *
Food intake	-	-	-	20.69	19.58	19.58	17.74	17.71	17.80
(g/day)				±0.82	±0.29	±0.28	±1.09	±0.36	±0.12
Water intake	-	-	-	20.12	20.45	20.27	20.52	21.58	20.30
(ml/day)				±1.27	±1.35	±1.02	±1.12	±0.96	±1.59
KW	-	-	-	-	-	-	2.85	2.32	2.24
(g)							±0.07	±0.01 *	±0.04 *
KW/BW	-	-	-	-	-	-	0.61	0.59	0.59
(* 100)							±0.02	±0.01	±0.01

Values are mean ± SEM (*n* = 6 each). Veh: vehicle-treated group; BPA: BPA-treated group; BPA+NAC: BPA plus NAC-treated group; BW: body weight; KW: kidney weight. * *p* < 0.05 vs. Veh within the same week. ^‡^ *p* < 0.05 vs. their respective values in week 0.

## Data Availability

The data presented in this study are available on request from the corresponding author.
