# Correction: Kobroob et al. Effectiveness of N-Acetylcysteine in the Treatment of Renal Deterioration Caused by Long-Term Exposure to Bisphenol A. *Biomolecules* 2021, *11*, 655

**DOI:** 10.3390/biom13121781

**Published:** 2023-12-12

**Authors:** Anongporn Kobroob, Wachirasek Peerapanyasut, Sirinart Kumfu, Nipon Chattipakorn, Orawan Wongmekiat

**Affiliations:** 1 Division of Physiology, School of Medical Sciences, University of Phayao, Phayao 56000, Thailand; anongporn.ko@up.ac.th; 2 Renal Physiology Unit, Department of Physiology, Faculty of Medicine, Chiang Mai University, Chiang Mai 50200, Thailand; wachirasek.pee@mahidol.ac.th; 3 Cardiac Electrophysiology Research and Training Center, Department of Physiology, Faculty of Medicine, Chiang Mai University, Chiang Mai 50200, Thailand; bc_nart@hotmail.com (S.K.); nipon.chat@cmu.ac.th (N.C.)

The authors would like to replace Figure 2 of the following published paper [1]. The new Figure 2 is attached below.

The authors apologize for any inconveniences caused and state that the scientific conclusions of the paper are unaffected.

## Figures and Tables

**Figure 2:**
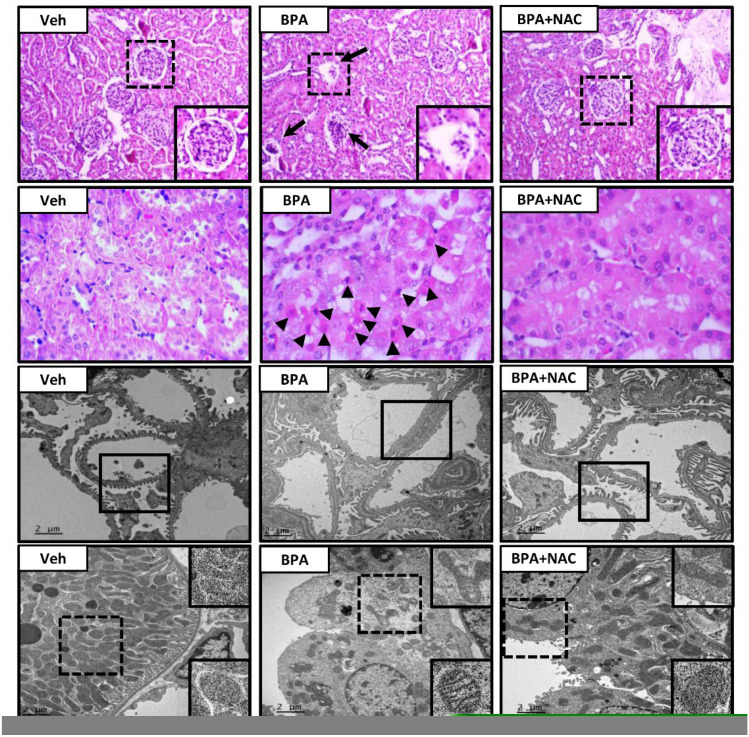
Histopathological changes following long-term BPA exposure and NAC treatment. The first and second panels show kidney sections stained with hematoxylin and eosin (H&E, 10× and 40×, respectively). The third and last panels show transmission electron micrographs of glomerulus and renal tubules, respectively (original magnification: 3000×). Veh: vehicle-treated group; BPA: BPA-treated group; BPA + NAC: BPA plus NAC-treated group. Arrows and arrowheads show abnormal glomerulus and apoptotic cells, respectively. The inserted frame is enlarged from the dashed area. The squares within the third panel highlight the morphology of the podocytes, especially the podocyte effacement in the BPA-treated group.
